# Establishing career competencies for the fourth industrial revolution: a qualitative study of expert perspectives in South Africa

**DOI:** 10.3389/fpsyg.2026.1823256

**Published:** 2026-05-07

**Authors:** Sanelisiwe Mtshali, Nelesh Dhanpat

**Affiliations:** College of Business and Economics, Department of Industrial Psychology and People Management, University of Johannesburg, Johannesburg, South Africa

**Keywords:** career competencies, career psychology, competencies, fourth industrial revolution, South Africa

## Abstract

**Introduction:**

The Fourth Industrial Revolution (4IR) is reshaping work through the convergence of physical, digital and biological domains, yet career psychology lacks empirical research on the competencies required for general workforce success in this context. Existing frameworks are occupation-specific and Western-centric, ill-suited to South Africa’s conditions of 31.4% unemployment, skills mismatches and digital literacy shortages. This study aimed to establish career competencies for effective career management in South Africa’s 4IR workforce and to elicit expert insights on emerging competency demands.

**Methods:**

A qualitative design was employed within a pragmatist, constructivist framework. Semi-structured interviews were conducted with 14 purposively selected South African experts in career development, competency assessment and organisational transformation, holding postgraduate qualifications and representing six industry sectors with 5 to 25 years of relevant experience. Data were analysed using Braun and Clarke’s six-step reflective thematic analysis supported by ATLAS.ti (version 23, Windows).

**Results:**

Analysis of 6,480 coded references identified six themes across 28 sub-themes: interpersonal skills (23.1%), mindset (18.3%), self-direction and agency (17.4%), change agility (17.3%), technology and data (12.1%) and contextual competencies (11.8%).

**Discussion:**

The findings extend the intelligent careers framework to 4IR demands in a developing economy. The prominence of human-centred over technical competencies challenges technology-dominant assumptions. Novel contributions include cognitive load management, boundary-setting and work-life integration as discrete career competencies, contextualised within South Africa’s structural constraints. The framework offers actionable guidance for career counsellors, human resource practitioners and policymakers developing 4IR-ready workforces in resource-constrained contexts.

## Introduction

The Fourth Industrial Revolution (4IR) is reshaping work through the convergence of physical, digital and biological technologies, fundamentally altering career trajectories, employment structures and the competencies required for sustained employability (Schwab, 2015, 2017). Despite growing recognition of this transformation (Hirschi, 2018), career psychology lacks empirical research on the specific career competencies needed for success in the 4IR, particularly for general workers in emerging economies such as South Africa (Habanabakize, 2024). Prevailing assumptions about technology dominance remain largely untested in diverse organisational and cultural settings (Habanabakize, 2024), creating a gap that poses practical challenges for career psychologists, human resource professionals, educators and policymakers requiring evidence-based guidance for developing a 4IR-ready workforce.

Three interconnected problems necessitate this study. First, existing career competency frameworks lack generalisability across occupations. Studies have focused on specific professional groups, including accommodation managers in tourism (Wessels et al., 2017), radiographers (Mung’omba and Botha, 2017), entrepreneurs (Sinyolo and Mudhara, 2018) and leaders (Munsamy et al., 2023), leaving career competencies for the general 4IR workforce unexplored (Hirschi, 2018). Second, predominant career competency models are largely grounded in Western contexts (Akkermans et al., 2013; Francis-Smythe et al., 2012), with evidence drawn predominantly from the United States (Kuijpers and Scheerens, 2006), the United Kingdom (Francis-Smythe et al., 2012) and Australia (Sargent and Domberger, 2007). Career development cannot be divorced from context (Einarsdóttir et al., 2010) or culture (Leong and Pearce, 2014), and South Africa’s distinct socio-cultural (Maree, 2019), economic and technological landscape (Watson and McMahon, 2018) makes direct transplantation of these models problematic (Savickas, 2005; Sullivan and Baruch, 2009). Third, South Africa’s unemployment rate of 31.4% (Statistics South Africa, 2025) and youth unemployment rate of 46.1% (Statistics South Africa, 2025) intersect with significant skills mismatches (Seti et al., 2025), historical inequalities affecting access to education and technology (National Planning Commission, 2012; Presidential Commission on the Fourth Industrial Revolution, 2020), and longstanding shortages of science, technology, engineering and mathematics (STEM) and digital literacy skills [Department of Basic Education (DBE), 2020; Schofield, 2014]. These structural conditions create an urgent need for 4IR-specific, contextually appropriate competency frameworks (Khoza, 2025).

The primary objective of this study was to establish career competencies for effective career management in South Africa’s 4IR workforce. The secondary objective was to elicit and explore expert insights on emerging career competencies in the 4IR context.

### The 4IR and its impact on work

Schwab (2015) identified three dimensions distinguishing the 4IR from preceding industrial revolutions: velocity, referring to unprecedented speed of change driven by interconnected technologies; breadth, encompassing simultaneous disruption across all sectors; and depth, involving systemic transformation of production, management and governance. A key differentiator is that computers and machines collaborate more independently through artificial intelligence (AI), rather than requiring constant human intermediation (Kim, 2019). AI systems employ algorithms enabling autonomous decision-making without direct human intervention (Riedl, 2019; Surden, 2019), shifting the human role from operator to collaborator and necessitating fundamentally different competencies from those sufficient for earlier industrial contexts (McGrath et al., 2019).

Pfohl et al. (2015) identified seven characteristics constituting the 4IR with direct career competency implications: digitalisation, requiring digital literacy and data interpretation; automation, demanding human-AI collaboration capabilities; transparency, necessitating ethical reasoning and algorithmic accountability; mobility, requiring self-management and digital communication; modularisation, demanding adaptability and systems thinking; network collaboration, requiring platform navigation and distributed teamwork; and socialisation with intelligent systems, necessitating new forms of human-machine communication. Collectively, these characteristics establish that effective 4IR career management requires competencies extending significantly beyond those sufficient for previous industrial eras.

The centrality of interpersonal competencies in this study also has relevance beyond the 4IR context. The Fifth Industrial Revolution (5IR) explicitly recenters human values within technological system (Ziatdinov et al., 2024) moving from the productivity-driven automation logic of the 4IR towards human-technology collaboration oriented around well-being and societal benefit (Maddikunta et al., 2022). The competencies identified here, particularly empathy, virtual collaboration, boundary-setting and work-life integration, are foundational to this emerging paradigm. The framework therefore offers utility not only for 4IR workforce development but as a foundation for the human-centred competency demands of the 5IR transition.

### Job transformation and employment polarisation

The World Economic Forum (2019) emphasises that 4IR strategies should leverage the broader range of value-creating activities humans uniquely perform, rather than focusing solely on automation-based cost reduction (Willcocks, 2024). Research confirms that organisations adopting augmentation strategies, through which technology enhances rather than replaces human workers, achieve superior outcomes to those focused solely on automation (Lei and Kim, 2025; Ren and Chowdhury, 2025).

In the South African context, 4IR adoption presents significant economic potential, with projected productivity increases of 2.1%, average income growth of 2.5% and GDP expansion of 3.5% (Magwentshu et al., 2019). These projections suggest net job creation of 4.5 million positions by 2030, yielding a net gain of 1.2 million jobs when accounting for displacement (Magwentshu et al., 2019). However, this net positive outcome obscures significant sectoral polarisation: growth concentrates in healthcare, construction and business services, whilst manufacturing, administrative roles and retail positions face substantial decline (Magwentshu et al., 2019). Displacement risk is highest among roles requiring physical, manual, routine and transactional tasks (Phillips et al., 2018; World Economic Forum, 2018), which constitute the majority of South African employment. This pattern requires that 4IR competencies enable occupational mobility, as individuals will increasingly need to transition between declining and growing sectors (Sposato, 2025).

The 4IR has also accelerated the shift from traditional permanent employment to non-standard work arrangements encompassing gig economy platforms, freelancing, short-term contracts and hybrid remote models (Postepska, 2022). These arrangements operationalise the theoretical constructs of boundaryless and protean careers (Arthur and Rousseau, 1996), yet create complex psychological demands: whilst autonomy increases job satisfaction for some workers, income instability and absence of employment benefits affects others, particularly those entering non-standard arrangements through necessity rather than choice (Bech-Wysocka et al., 2025). Boundary erosion between work and personal time produces adverse effects on mental health and performance (Mascarenhas et al., 2025), reinforcing the need for self-management and boundary-setting as career competencies.

### Structural barriers in the South African context

South Africa’s particular structural conditions create unique challenges for 4IR career development that Western frameworks do not adequately address. Three interrelated barriers constrain individuals’ capacity to develop and deploy 4IR competencies.

The Organising Framework for Occupations (OFO), which categorises occupations through task-based classifications, proves increasingly problematic in a labour market demanding adaptable skills rather than job-specific preparation (Hattingh, 2016). By prioritising occupation-specific preparation over transferable competency development, the OFO produces individuals trained for positions that may no longer exist whilst inadequately preparing them for emerging opportunities (Hattingh, 2017). Career guidance has traditionally matched individuals to specific jobs, but 4IR contexts require continuous learning, flexibility and adaptation to new roles (Gartoumi and Tekouabou, 2024).

Infrastructure and connectivity constraints compound this challenge. Africa lags behind developed and emerging economies in the infrastructure, technology and education necessary for 4IR participation (Ndung’u and Signé, 2020), creating a two-tier system in which some individuals access global opportunities through digital platforms whilst others cannot participate at all (Marchal et al., 2007). The South African Gastrow (2018) emphasises that 4IR transformation in Africa involves not merely technological implementation but also addressing socio-political dimensions, requiring simultaneous attention to infrastructure development and competency enhancement.

High unemployment further constrains the applicability of Western career theories emphasising choice, mobility and self-direction (Sultana, 2024). Concepts such as protean careers, emphasising values-driven choice, or boundaryless careers, emphasising mobility, become aspirational rather than practical frameworks when employment opportunities remain scarce (Maree, 2020). In contexts characterised by high unemployment, individuals prioritise job security and adequate compensation over value alignment or career exploration (Maree, 2020), making survival rather than self-actualisation the primary career motivation. AI-driven employment processes, including automated CV screening and online job seeking, create additional inclusion challenges, as candidates with limited digital resources face systematic exclusion (George et al., 2025).

### Socio-political responses revealing competency gaps

The socio-political responses to 4IR disruption in South Africa reveal systemic failures in competency preparation. The introduction of Uber exemplifies inadequate transition management: rather than prompting questions about skills acquisition or career adaptation, the service faced violent resistance from traditional taxi operators, resulting in fatal confrontations (Henama and Sifolo, 2017). This response reflects not only technical skills deficits but also the absence of meta-competencies enabling individuals to recognise disruption, identify alternative opportunities and manage career transitions without perceiving them as existential threats. Similarly, the South African Society of Bank Officials, representing 73,000 members, planned what would have been the largest banking strike in 99 years in response to 4IR-related job losses (ENCA, 2019), demonstrating that even skilled, formally educated workers felt unprepared for technological transformation. Labour unions have broadly supported workers opposing 4IR changes, generating workplace instability and industrial action (Mhene, 2019; Parliamentary Monitoring Group, 2019). These responses are symptoms of inadequate career preparation rather than inevitable consequences of technological change.

### Theoretical limitations and the need for a new framework

Existing career theories prove inadequate for South African 4IR contexts in three fundamental ways (Hirschi, 2018). First, they lack operational specificity: protean and boundaryless career theories identify broad orientations such as adaptability and self-direction without specifying the behavioural and cognitive competencies that enable individuals to enact these orientations in 4IR settings. This study addresses that gap not by proposing a fixed prescriptive model, which would risk rapid obsolescence in a context of continuous technological change, but by offering an empirically grounded, contextually situated competency framework that is open to iterative revision. Second, they lack 4IR-specific operational definitions: terms such as adaptability, digital literacy and self-management appear throughout the literature yet remain insufficiently operationalised for contexts involving human-AI collaboration and platform-based employment (Ologunoye et al., 2025). Third, they assume Western contexts inappropriate for developing economies (Arthur and Rousseau, 1996; Hall, 2002), given South Africa’s infrastructure constraints, employment scarcity and rigid certification systems.

The intelligent careers framework (Arthur et al., 1995; DeFillippi and Arthur, 1994), which categorises career competencies into knowing why, knowing how, knowing whom, knowing what, knowing when and knowing where, provides useful organisational structure aligned with 4IR employment patterns of increased mobility and individual responsibility (Jones and Defillippi, 1996). It incorporates both individual and contextual factors and offers conceptual distinction from job-specific skills, making it suitable for identifying transferable competencies across occupations (DeFillippi and Arthur, 1994). However, the framework predates widespread 4IR technologies, potentially limiting its comprehensiveness regarding AI collaboration and algorithmic literacy (Bouwmans et al., 2024; Coetzee et al., 2021). The current literature presents component theories, contextual challenges and potential skill lists but lacks an integrated, validated and holistic framework that is comprehensive, specific and contextually appropriate for South Africa (Borrageiro and Mennega, 2023; Gartoumi and Tekouabou, 2024).

This study addresses that gap. Employing the intelligent careers framework as an organisational structure whilst remaining empirically open to emergent competencies, it consults South African experts with direct experience of 4IR workplace transformation to identify competencies accounting for infrastructure limitations, economic constraints and socio-political dynamics.

## Materials and methods

### Research approach and philosophy

This study adopted a pragmatist research paradigm (Creswell and Plano Clark, 2018), prioritising practical knowledge generation suited to the complexity of the research problem. A relativist ontological stance acknowledged that reality is socially constructed and context-dependent (Denzin and Lincoln, 1994), which is appropriate given that 4IR career competency requirements differ substantially across industries, organisational structures and the socio-economic circumstances characteristic of South Africa (Somekh and Lewin, 2005). A constructivist epistemological position informed the qualitative design, recognising that knowledge emerges through researcher-participant interaction rather than existing as objective, pre-given truth (Denzin and Lincoln, 1994). This justified semi-structured interviews as a process of collaborative meaning-making (Vygotsky, 1978), enabling context-specific competency insights to surface that predetermined instruments could not capture.

A qualitative design employing expert purposive sampling was used to establish 4IR career competencies from the perspectives of credentialed practitioners with direct professional experience of workforce transformation. This approach is appropriate for framework development in emerging research areas (Malterud et al., 2016; Patton, 2015) but does not claim to represent the lived career experience of the broader South African workforce, which is addressed as a limitation and a direction for future research.

### Sample and procedure

A non-probability purposive sampling approach was employed to identify participants with specialised knowledge and direct professional experience relevant to 4IR career competencies (Patton, 2015). Purposive sampling is appropriate when the objective is to generate rich, contextually grounded insights rather than statistical generalisation (Palinkas et al., 2015), and expert sampling is particularly well suited to studies concerned with emerging constructs in applied settings, where practitioners’ situated knowledge yields insights unavailable from general population samples (Malterud et al., 2016).

Three complementary techniques were combined. Criterion sampling established mandatory inclusion standards: a minimum honours degree (NQF Level 8) in industrial and o rganisational psychology, psychometrics, human resource management or a related discipline; at least five years of direct professional experience with career competencies, workforce development or 4IR implementation from 2016 to the present; active employment in an advisory, leadership, technical or research role involving competency assessment or organisational transformation; and evidence of professional engagement with technological transformation or digital workplace strategy. Maximum variation sampling ensured diversity across industry sectors, organisational contexts and professional roles (Patton, 2015). Snowball sampling supplemented recruitment by leveraging participants’ professional networks to access qualified individuals not reachable through direct outreach (Noy, 2008), with all nominated candidates assessed against the same inclusion criteria.

A target of 12 to 16 participants was established based on methodological guidance for expert interview studies (Malterud et al., 2016; Morse, 2015). Information power is elevated when participants are credentialed experts addressing focused research questions, meaning fewer participants can yield theoretically rich data (Malterud et al., 2016). Guest et al. (2020) found that 92% of themes emerge by the twelfth interview in homogeneous expert samples. The final sample of 14 participants falls within this range. Saturation was monitored systematically after each interview and confirmed when three successive interviews produced no novel competency categories; three confirmatory interviews were conducted beyond initial saturation, reached at Interview 11, following best practice recommendations (Hennink and Kaiser, 2020).

Of 25 experts initially contacted, 18 accepted invitations and four subsequently withdrew due to scheduling conflicts, yielding a final sample of 14 participants. Data collection took place between 23 September and 5 November 2024. Participant demographic characteristics are presented in Table 1.

**Table 1:** Demographic information of qualitative interview participants.

Participant	Gender	Age range	Qualification	Industry	Years (competencies/4IR)	Job title
P1	Female	30–39	Master’s	Professional services	9 years	Head of consulting
P2	Male	30–39	PhD	Professional services / higher education	11 years	Senior consultant and IOP board member at a higher learning institution
P3	Female	30–39	Master’s	Manufacturing and mining	6 years	Organisation design manager
P4	Female	40–49	Master’s	Energy and utilities	10 years	Senior advisor, psychometrics and skills assessment
P5	Female	40–49	Master’s	Banking and finance	14 years	Lead organisational design and assessments
P6	Male	30–39	PhD	Higher education	5 years	Lecturer
P7	Female	30–39	Master’s	Higher education	7 years	Senior organisational psychologist, students’ counselling
P8	Male	40–49	PhD	Higher education	25 years	Senior lecturer
P9	Male	50–59	PhD	Higher education	10 years	Professor
P10	Female	40–49	Master’s	Professional services / higher education	20 years	Professor and head of people development
P11	Female	40–49	Master’s	Financial services	9 years	Group head of learning
P12	Male	30–39	PhD	Higher education	7 years	Lecturer
P13	Female	40–49	PhD	Professional services and higher education	10 years	Independent practice and senior lecturer
P14	Female	40–49	PhD	Transport and logistics	10 years	Human resource manager

The sample was predominantly female (71.4%), with most participants aged 30 to 49 years (*n* = 13) and one aged 50 to 59 years. All held postgraduate qualifications, with equal representation between Master’s degrees (*n* = 7, 50%) and doctoral qualifications (*n* = 7, 50%). Sectoral coverage was broad: higher education was most represented (*n* = 6), followed by professional services (*n* = 4), financial services (*n* = 2), and manufacturing, mining, energy, transport and logistics (one participant each), with several participants holding dual roles across sectors. Professional experience ranged from 5 to 25 years, with the majority (*n* = 8, 57.1%) having between 5 and 10 years, three between 11 and 15 years, and three between 16 and 25 years.

Participants were well suited to the study’s research questions. All held direct professional experience with career competencies and had navigated organisational change associated with the 4IR. Their advanced qualifications enabled conceptually precise articulation of complex competency constructs, and sectoral diversity ensured that findings reflected experiences across multiple industries undergoing 4IR transformation.

### Data collection

Semi-structured interviews were used to collect data. This method was appropriate for three reasons: the exploratory nature of the study required flexibility to uncover unforeseen insights (Ruslin et al., 2022); it allowed participants to articulate their expertise in their own words whilst ensuring consistent coverage of key topics (McGrath et al., 2019); and flexibility was essential for examining a complex construct across participants with varied professional backgrounds (DeJonckheere and Vaughn, 2019).

An interview guide of 16 open-ended questions was developed through a systematic review of the career competencies and 4IR literature (Creswell and Creswell, 2017). Core questions included: “What are the key career competencies essential for the 4IR workforce in South Africa?” and “What personal attributes are essential for today’s workforce?” A formal interview protocol organised the sequence of questions whilst allowing adaptive probing and maintaining interviewer neutrality (DeJonckheere and Vaughn, 2019; Shoozan and Mohamad, 2024). All 14 interviews were conducted via Microsoft Teams to enable geographic representation across South African provinces (Archibald et al., 2019). Interviews lasted between 37 min and 1 h 15 min, were audio-recorded with participant consent, and transcribed verbatim (Given, 2008; McMullin, 2023). Data collection continued until saturation was confirmed (Lim, 2025).

### Data trustworthiness

Trustworthiness was established through the four criteria established by Lincoln and Guba (1985). Credibility was addressed through prolonged engagement via comprehensive interviews, member checking through participant verification of interview summaries, and triangulation across diverse industries and professional roles (Korstjens and Moser, 2018). Dependability was ensured through systematic documentation of all research procedures, preservation of raw data and audio recordings, and transparent coding records tracking the reduction from 232 initial codes to 94 consolidated codes (Forero et al., 2018). Transferability was supported through detailed descriptions of participant characteristics, industry representation and the South African socio-economic context, enabling readers to assess applicability to their own settings (Tenny et al., 2022). Confirmability was achieved through a reflexive journal documenting researcher assumptions throughout the analytical process, peer review of analytical procedures, and the grounding of all conclusions in direct participant quotations (Johnson et al., 2020).

### Data analysis

Braun and Clarke's (2012, 2022) six-step reflective thematic analysis framework was employed to analyse the interview data, supported by ATLAS.ti (version 23, Windows) for systematic coding and code co-occurrence analysis. Thematic analysis is appropriate for qualitative research because it organises and describes data in rich detail whilst enabling the identification of patterns across a complex dataset (Braun and Clarke, 2021).

In Step 1, the researcher familiarised themselves with the dataset through repeated reading of all transcripts and recording of initial observations (Creswell, 2013; Merriam, 2009). In Step 2, inductive coding via ATLAS.ti generated 232 unique codes capturing technical skills, non-technical competencies, behavioural indicators and contextual factors. In Step 3, codes sharing conceptual characteristics were consolidated, reducing the total to 94, which were then organised into candidate themes. In Step 4, provisional themes were reviewed for coherence with coded extracts and representativeness of the full dataset, following Patton's (1990) and Byrne's (2022) dual criteria of internal homogeneity and external heterogeneity. In Step 5, each theme and sub-theme received a clear name and definition. Six primary themes and 25 sub-themes emerged: (a) self-direction and agency, (b) change agility, (c) interpersonal skills, (d) technology and data, (e) mindset, and (f) contextual competencies. In Step 6, the analysis was translated into a report integrating analytical narratives with participant quotations and situating findings within the existing literature (Creswell, 2014; Miles et al., 2014). A frequency table derived from ATLAS.ti’s code co-occurrence function is included in the findings to substantiate empirical validity.

Quotations presented in the Results section were selected on the basis of illustrative clarity and thematic centrality rather than equal participant representation. All 14 transcripts contributed to the full coding and frequency analysis; the selection of specific verbatim extracts prioritised the most precisely articulated expressions of each sub-theme. Additional quotations from participants not initially quoted have been incorporated to ensure broader representational transparency.

### Ethical considerations

Ethical clearance was obtained from the Department of Industrial Psychology and People Management’s Research Ethics Committee (IPPM-2020-454(D)) prior to data collection. All participants provided written informed consent and were advised that participation was voluntary, that they could withdraw at any stage without consequence, and that their confidentiality would be protected. All identifying information was removed from transcripts and participants are referred to by code throughout the study. Audio recordings and transcripts were stored securely with access restricted to the research team.

## Results

### Overview of thematic structure

Thematic analysis of the 14 expert interviews identified six main competency themes comprising 25 sub-themes, based on 6,480 coded references. Table 2 presents the full thematic structure with sub-theme frequencies.

**Table 2:** Frequencies for themes and sub-themes.

Theme	Sub-theme	Frequency	Theme total
Theme 1: Self-direction and agency	Proactivity	182	1,126
	Open-mindedness	175	
	Goal-setting	176	
	Continuous learning	162	
	Broadening and diversifying knowledge and skill	295	
	Self-evolution	136	
Theme 2: Change agility	Adaptability	289	1,122
	Resilience	243	
	Emotional intelligence	225	
	Work-life integration	199	
	Boundary-setting	166	
Theme 3: Interpersonal skills	Communication skills	209	1,495
	Collaboration	206	
	Ethical behaviour	228	
	Empathy	201	
	Networking	203	
	Teamwork	198	
	Virtual and online engagements	250	
Theme 4: Technology and data	Digital literacy	144	786
	Data analysis	135	
	Technological adaptability/knowledge	125	
	AI proficiency	131	
	Professional social media	251	
Theme 5: Mindset	Critical thinking	158	1,186
	Complex problem-solving	141	
	Creative and innovative thinking	360	
	Decision-making	180	
	Cognitive load management and information processing	122	
	Dynamic understanding	225	
Theme 6: Contextual competencies	Diversity and inclusion	200	765
	Cultural awareness and sensitivity	212	
	Business and market acumen	353	
	Contextual business intelligence	131	
Total		6,480	6,480

Frequency counts are derived from ATLAS.ti code co-occurrence analysis across 14 expert interviews.

### Theme 1: Self-direction and agency

Self-direction and agency received 1,126 coded instances (17.4% of total coded references), reflecting the importance of personal ownership and proactive behaviour in dynamic work environments. This theme encompasses six sub-themes: proactivity, open-mindedness, goal-setting, continuous learning, broadening and diversifying knowledge and skills and self-evolution.

#### Proactivity

Participants consistently described proactivity as taking charge of one’s own career and development without external prompting.

“People who take responsibility for their careers on their own … take proactive control … are much more employable … people who take proactive control of their work tend to be happier; in other words, my career is not happening to me.” (P2)

“It’s not an HR responsibility, it’s a person’s responsibility … if you can create a learning culture within individuals … you take them to the water, but you cannot make them drink.” (P1)

#### Open-mindedness

Open-mindedness was described as extending beyond passive acceptance to active engagement with new technologies and methodologies, linked directly to growth mindset and career sustainability.

“If we are not open for the new and do not have a growth mindset, our businesses and careers will stagnate and eventually fade away. Open-mindedness in the Fourth Industrial Revolution is essential for embracing new technologies and evolving with industry demands.” (P4)

“Open-mindedness is tied to a willingness to embrace new technologies and methodologies. In the Fourth Industrial Revolution, it’s about learning continuously and being ready to pivot as the environment shifts.” (P7)

#### Goal-setting

Participants emphasised the alignment of personal goals with organisational objectives as both a protective mechanism against career stagnation and a driver of advancement.

“See what the needs of the organisation are and really know where to pitch it … strategic focus, when linked to the goals of the organisation, really takes it to the next level.” (P9).

“Having the freedom to structure my job and my career in a way that I prefer to do it based on what I see is important in the organisation or in the world of work.” (P2)

“You do not know what you are trying to achieve, so this idea of personal effectiveness, being very clear on your own goals and where the job you are doing fits in to help you get there, will also help you to be more resilient.” (P1)

#### Continuous learning

The nature of continuous learning was described as having evolved significantly, characterised by accelerated pace and cyclical processes of learning, unlearning and relearning.

“It means being agile enough to speak about learning, relearning, and unlearning … you need to learn something, but maybe you need to unlearn it and really learn new things.” (P5)

“You got to create the hunger or the need for people to keep on learning and to keep on developing.” (P1)

“In the 4IR, the ability to learn at pace is non-negotiable. We actively coach people to identify what to stop, start, and continue in terms of their learning habits” (P10)

#### Broadening and diversifying knowledge and skills

Participants highlighted a shift away from single specialisation towards a broader, more adaptive approach to skill development, encompassing human-machine collaboration.

“The core technical skill for the 4IR would be human-machine collaboration. Humans need to learn how to use machines and new tools. This calls for broadening the skill set to include technical and cognitive skills alongside traditional roles.” (P6)

“Organisations are realising that it is not about having a single specialised skill anymore. Employees must possess a broader range of competencies to remain relevant in dynamic industries.” (P8)

#### Diverse knowledge

Diverse knowledge was identified as enabling innovation through the combination of insights from different fields.

“Organisations will require employees to build networks across industries and organisations, accessing diverse knowledge pools. In the 4IR, having insights from different domains is essential for innovation and addressing complex problems.” (P2)

“Knowledge transfer programmes are critical for retaining talent and exposing employees to diverse perspectives. The 4IR requires this diversity to foster creativity and adaptability.” (P3)

#### Self-evolution

Self-evolution was described as extending beyond continuous learning to encompass a fundamental reimagining of career identity, moving from deep specialisation towards broader skill applications across multiple contexts.

“Individuals who take charge of their careers through continuous learning and self-evaluation tend to thrive. In the Fourth Industrial Revolution, self-evolution is no longer optional; it’s the difference between staying relevant and becoming obsolete.” (P2)

“The research is showing that organisations will become smaller in the future … individuals will be required to build networks with multiple organisations at the same time to remain employable.” (P2)

### Theme 2: Change agility

Change agility received 1,122 coded instances (17.3% of total coded references). This theme encompasses five sub-themes: adaptability, resilience, emotional intelligence, work-life integration and boundary-setting.

#### Adaptability

Adaptability was described as encompassing not only technological change but broader cognitive and behavioural flexibility, linked directly to career sustainability.

“Adaptability is absolutely critical, and change has become the norm. People who are not flexible, who cannot deal with ambiguity and unpredictability, really struggle.” (P2)

“The Fourth Industrial Revolution is basically upon us and asking us to adapt to the working situation and to be able to adapt all the time … not staying behind.” (P8)

#### Resilience

Resilience was highlighted as both a personal survival strategy and a career sustainability requirement.

“Having the capability to cope with pressures, be resilient, and have positive coping mechanisms has become more critical than ever before. The Fourth Industrial Revolution requires us to manage stress effectively in order to stay productive and engaged in complex environments.” (P2)

“Ambiguity and complexity have become the norm. You need to be able to manage in uncertain and dynamic situations where traditional problem-solving methods might not apply. This calls for resilience, creativity, and quick thinking.” (P5)

“We see a direct correlation between low digital confidence and burnout. The skills gap is not just a performance issue, it actively erodes people’s capacity to cope” (P11)

#### Emotional intelligence

Emotional intelligence was identified as fundamental to the 4IR era, manifesting across personal emotional management, empathetic leadership and effective interpersonal interactions.

“In the 2030s and beyond, the person who would be needed in terms of leadership competencies is the person with high emotional intelligence, looking at empathy, leading with empathy.” (P1)

“Emotional intelligence is more relevant than ever in the Fourth Industrial Revolution. It starts with understanding yourself and extends to managing interactions with others effectively in a rapidly changing technological landscape.” (P8)

#### Work-life integration

A significant shift was noted from traditional concepts of work-life balance to a more fluid work-life integration model, driven by technology changing the boundaries between professional and personal spheres.

“Work-life integration is what they should have, because if you work from home, you can work 24/7 … the ability to work under pressure, stress tolerance, is very important in terms of work-family integration.” (P9)

#### Boundary-setting

Participants identified a critical tension between organisational demands for constant connectivity and individual needs for mental health protection.

“You must also be able to have different boundaries in your life … you will burn yourself out in the 4IR because you can work constantly 24/7.” (P9)

“There’s also a need for us to set boundaries to say it ends here … if you do not manage it properly, you stand to have mental health issues.” (P3)

### Theme 3: Interpersonal skills

Interpersonal skills gathered 1,495 coded references (23.1% of total coded references), the highest of all themes. This theme encompasses seven sub-themes: communication skills, collaboration, ethical behaviour, empathy, networking, teamwork and virtual and online engagements.

#### Communication skills

The 4IR was described as having transformed communication from primarily face-to-face interactions to diverse digital modes, requiring clarity, conciseness and multi-platform adaptation.

“Regardless of the mode of communication, we need to adapt the communication to suit the mode as well.” (P3)

“When you write something, it needs to be as clear and concise as possible. Instead of writing a 20-page report, how about you write a five-page report but write it so clearly and so articulately that the audience gets the message.” (P5)

#### Collaboration

Collaboration was described as having evolved beyond simple task-sharing to encompass knowledge integration, cross-functional expertise and the synergy between human and technological capabilities.

“Being aware of the value that you can offer as a human employee, in combination with the technology, where you sort of feed off each other.” (P2)

“Collaborating with technology, basically human-machine interaction, is being able to tackle problems because of the nature of the problems that we are going to be facing in the context of the 4IR world.” (P6)

#### Ethical behaviour

Ethical behaviour emerged as requiring integration with technological advancement, with participants emphasising governance, responsible innovation and the coexistence of ethics and forward-thinking.

“Ethical responsibility must coexist to anticipate and solve challenges effectively, especially in a rapidly changing environment.” (P2)

“The ability to govern whilst fostering a culture of innovation reflects the complex interplay of ethics and forward-thinking in modern industries.” (P4)

#### Empathy

Empathy was described as having evolved from a basic interpersonal skill to a strategic organisational tool, serving as a mediator between technological advancement and human well-being.

“When you tell the work settings that empathy is a critical skill that you would need to deliver on your goals and processes … when you are looking at what is sitting in front of us currently, you would understand why empathy should be elevated.” (P1)

“If we are not empathetic, there will not be proper collaboration, proper teamwork … for all those things to work well, we need good communication skills.” (P12)

#### Networking

Networking was described as having transformed from traditional face-to-face relationship-building to a multifaceted approach spanning digital and physical spaces.

“If you do not have the capability to build relationships not just within your own organisation, but across industries, across levels, across different organisations, you will really struggle.” (P2)

“Visibility becomes key … networking and building relationships becomes key.” (P3)

#### Teamwork

Teamwork was identified as having shifted from traditional hierarchical, co-located structures to fluid, self-managing virtual teams.

“I think productivity and working without much supervision is necessary because, as we work in different geographies without our actual teams close to us, it needs you to have that skill.” (P2)

“In the world of agile, they are starting to say everyone is a leader and therefore people should be able to self-manage.” (P9)

#### Virtual and online engagements

Virtual and online engagements were identified as requiring more than technical proficiency, demanding a fundamental rethinking of professional interaction and the interpretation of virtual social cues.

“Being able to read somebody in a virtual context is very important … virtuality is an important competency that somebody should look at.” (P2).

“The fact that people are working remotely more and more means that the way you create cohesion in teams needs to be different.” (P1)

“One of the things we had to actively teach during remote work was how to signal presence and build trust without being physically visible. That is a new skill entirely.” (P10)

### Theme 4: Technology and data competencies

Technology and data competencies contained 786 coded references (12.1% of total coded references), reflecting the importance of digital competencies as foundational rather than dominant capabilities. This theme encompasses five sub-themes: digital literacy, data analysis, technological adaptability, AI proficiency and professional social media.

#### Digital literacy

Digital literacy was described as having evolved from basic computer skills to comprehensive capabilities enabling strategic technological integration and process optimisation.

“We need to educate our people that together with your physical technical skills, we will need people to fix those robots, we will need people.” (P1)

“What we see is the digitalisation and how you use and optimise technology and systems that you can introduce into your specific field.” (P4)

#### Data analysis

Data analysis was described as enabling informed decision-making, strategic planning and predictive capabilities, with participants noting the importance of combining qualitative and quantitative approaches.

“If you are working on talent management and data analytics in HR, there’s a need for you to be tech-savvy with different data analytics tools so that you can give insights into different areas of the business and the business can understand its current position and what it may look like in the future.” (P3)

#### Technological adaptability

Technological adaptability was characterised by a shift towards practical application and continuous learning over theoretical knowledge.

“The shift to a more agile workforce will expose people to diverse work types. It’s about adapting to practical applications rather than long theoretical courses.” (P1)

“Adapting to new technological tools is vital. You do not have to be a programmer to use these things, you just need to know the ones that are there.” (P6)

#### AI proficiency

AI proficiency was identified as an emerging competency reshaping work processes and organisational roles, including the potential emergence of dedicated AI prompt positions.

“ChatGPT prompt could become a position within organisations.” (P1)

“Learning AI tools at a faster pace is key, as the format and rate of learning differ significantly from the past.” (P5)

“AI proficiency enables automation, freeing up resources for more strategic focus and improving service efficiency.” (P7)

#### Professional social media

Professional social media was described as having a dual impact on personal branding and organisational effectiveness, requiring careful management to align personal presence with professional objectives.

“Your personal branding on social media is crucial. A single misstep can destroy potential professional relationships.” (P3)

“Organisations increasingly use professional platforms like LinkedIn to attract and retain talent.” (P1)

“We now explicitly coach clients on their LinkedIn strategy as part of career counselling. Digital visibility has become as important as qualifications for many roles” (P13)

### Theme 5: Mindset competencies

Mindset competencies emerged with 1,186 coded instances (18.3% of total coded references). This theme encompasses six sub-themes: critical thinking, complex problem-solving, creative and innovative thinking, decision-making and cognitive load management.

#### Critical thinking

Critical thinking was identified as a fundamental cognitive competency requiring both rapid decision-making and thorough analytical depth.

“Critical thinking. So we have to quickly evaluate the situation, see what decision to take and then implement it.” (P1)

“We do not ask anymore, and we just assume, or most of us take the information that we receive as is … you need to ask deeper questions beyond what your experience and knowledge allow you to do.” (P4)

#### Complex problem-solving

Complex problem-solving was characterised by increased speed of application, greater frequency of use and the need to function effectively with incomplete information.

“Problem-solving will never disappear … it is the complexity, the variables that come into play where contextual intelligence comes in.” (P4)

“We need people to be able to solve problems without having concrete information. Typical things include dealing with ambiguity and uncertainty.” (P1)

“I work with graduates who are technically strong but freeze when there is no clear answer. We need to build tolerance for ambiguity as a deliberate competency” (P11)

#### Creative and innovative thinking

Creativity was described as encompassing divergent thinking, the generation of novel solutions and the ability to think beyond current realities. Innovation was described as a strategic competency focused on practical implementation and organisational transformation rather than mere ideation.

“We need to think out of the box and be creative in our problem-solving … the ability to think what could be rather than what is.” (P4)

“Typical example, your Kodak, your Blackberries … those companies did not change with the times … they got lost and then other companies came that were innovative.” (P5)

“Problem-solving involves a form of innovation, and it involves a form of creativity. you cannot have one without the other.” (P1)

#### Decision-making

Decision-making was described as increasingly complex in the 4IR context, requiring a balance between human judgement and technological capabilities.

“How do we still uphold the people and the best interests of the people in this dynamic?” (P1)

#### Cognitive load management and information processing

Information processing and cognitive load management was identified as a distinct competency addressing the challenges of multiple simultaneous information streams and digital interruptions.

“I’m getting things on WhatsApp. I’m getting things on Teams. I’m getting emails. It’s not multitasking; it’s actually task switching in between, and it’s actually deteriorating my efficiency … how do we teach them to focus on one piece of work without interruption?” (P1)

“We are living with multiple sources of information and data and obviously different audiences.” (P5)

“Managing information overload is something we explicitly include in our digital wellness programmes now. It is not a soft issue it directly affects output quality” (P11)

### Theme 6: Contextual competencies

Contextual competencies comprised 765 coded references (11.8% of total coded references). This theme encompasses three sub-themes: diversity and inclusion, cultural awareness, business and market acumen.

#### Diversity and inclusion

Diversity and inclusion was identified as a strategic competency with a direct link to innovation, performance and organisational effectiveness.

“Interpersonal sensitivity is critical. Not everyone is in sync with the pace of change, and empathy becomes key to managing these transitions … sensitivity to how people adapt to change is essential for success in a culturally diverse environment.” (P3)

#### Cultural awareness

Cultural awareness was described as extending beyond basic sensitivity to encompass change management capabilities, societal impact understanding and sophisticated communication adaptation across cultural and digital contexts.

“Demonstrates understanding and respect for diverse cultural values, beliefs and communication styles … shows ability to adapt leadership and communication approaches to different cultural contexts.” (P8)

“Understanding the use of language not just as a language … but now even lingo.” (P5)

#### Business and market acumen

Business acumen was identified as a strategic competency linked directly to innovation, performance and navigating organisational change and requiring proactive anticipation of market dynamics and engagement with broader socio-economic stakeholder relationships.

“Sensitivity to how people adapt to change and managing these dynamics effectively is essential for success in a culturally diverse environment.” (P3)

“How is this impacting society, the 4IR, intercultural abilities of people, how to deal with different cultures in different environments and contexts?” (P9)

“It’s important for individuals to remain aware of what is happening in their industry so that they could potentially bring that into the organisation or, if their skills are no longer required, [take action accordingly].” (P2)

“As humans, we should not be complacent and must reskill ourselves in response to technological advancements to ensure we remain relevant and contribute meaningfully to the socio-economic landscape.” (P4)

## Discussion

### The primacy of interpersonal skills: challenging technology-dominant assumptions

The most significant finding is that interpersonal skills constituted the largest competency domain (23.1% of coded references), substantially exceeding technology and data competencies (12.1%). This directly challenges prevailing assumptions in the 4IR literature, which has tended to position digital and technical skills as the primary determinants of workforce readiness (Habanabakize, 2024; Phillips et al., 2018). The finding aligns instead with the World Economic Forum’s (2021) emphasis on the broader value-creating activities that humans uniquely perform, and with augmentation research confirming that organisations leveraging human capabilities alongside technology achieve superior outcomes to those focused on automation alone (Lei and Kim, 2025; Ren and Chowdhury, 2025).

Within interpersonal skills, empathy was identified as having evolved from a basic interpersonal capacity to a strategic organisational competency and a mediator between technological change and human well-being. This is consistent with Goleman’s (1998) foundational emotional intelligence work and with more recent evidence identifying empathy as a core leadership competency for complex, digitally mediated work environments (McMahon and Abkhezr, 2025). Participants explicitly linked empathy to the management of technological transitions, noting that without empathetic leadership, technological change generates resistance rather than adaptation. This connects to the socio-political cases described in this paper, in which violent responses to 4IR disruption reflected not only technical skills deficits but the absence of interpersonally skilled transition management (Henama and Sifolo, 2017; Sekaja et al., 2021).

The emergence of virtual and online engagements (250 coded references, the highest single sub-theme in interpersonal skills) as a distinct competency requiring more than technical proficiency reflects the post-COVID-19 normalisation of remote and hybrid work (Mascarenhas et al., 2025). Participants emphasised that reading social cues, maintaining cohesion and facilitating collaboration in virtual environments constitute a new competency domain not reducible to platform familiarity. This finding extends the boundaryless careers literature (Arthur and Rousseau, 1996) by specifying the interpersonal capabilities required to operate effectively within digitally mediated, geographically distributed work arrangements.

### Self-direction, agency and the protean career

Self-direction and agency (17.4% of coded references) encompassed proactivity, open-mindedness, goal-setting, continuous learning, broadening skill sets, diverse knowledge and self-evolution. These findings resonate strongly with the protean career model (Hall, 2002), which positions individual agency, values-driven adaptation and self-directed development as defining features of contemporary careers. The intelligent careers framework (Arthur et al., 1995; DeFillippi and Arthur, 1994) provides theoretical alignment through its knowing why and knowing how dimensions.

The sub-theme of self-evolution extended the continuous learning concept beyond what the existing literature has fully operationalised. Participants described a fundamental reimagining of career identity, moving from deep occupational specialisation towards broader, multi-contextual skill deployment across “collarless worker” arrangements. This reflects the theoretical trajectory from career as occupation to career as ongoing project of self-construction (Maree, 2020), and specifies concrete behavioural manifestations relevant to the South African 4IR context. The concept aligns with research on polyworking (Mkhize and Mkhize, 2024) and extends protean career theory by describing how individuals must reconceptualise career identity itself rather than merely adapting skills within an existing career framework.

Goal-setting received strong representation (176 coded references), with participants emphasising the alignment of personal goals with organisational objectives as a protective mechanism against stagnation. This operationalises the knowing why dimension in 4IR terms, adding specificity to Savickas (2005) career construction theory by positioning goal alignment not as a static orientation exercise but as a dynamic, iterative process requiring regular reassessment as technological demands evolve.

### Change agility: resilience, emotional intelligence and the work-life challenge

Change agility (17.3% of coded references) encompassed adaptability, resilience, emotional intelligence, work-life integration and boundary-setting. Adaptability received the highest single sub-theme frequency within change agility (289 coded references), consistent with career adaptability theory (Savickas and Porfeli, 2012). However, participants extended the concept beyond attitudinal orientations to include the cognitive flexibility required for human-AI collaboration and rapid skill transfer across technological domains.

Resilience (243 coded references) was described as both a personal survival strategy and a career sustainability requirement, consistent with Bimrose and Hearne’s (2012) identification of resilience as a foundational career management competency and with McMahon and Abkhezr’s (2025) emphasis on its growing importance in uncertain career contexts. The South African dimension adds specificity: participants noted that inadequate technological competence creates a vicious cycle in which skill gaps generate stress, which in turn undermines performance and further widens competency deficits, a pattern not addressed in Western resilience frameworks that assume foundational digital access and literacy.

The emergence of work-life integration and boundary-setting as distinct career competencies represents a significant conceptual contribution. Participants shifted the framing from the traditional work-life balance construct, which implies a static equilibrium, to work-life integration, reflecting the fluid and permeable nature of work and personal time in digitally connected environments (Mascarenhas et al., 2025). Boundary-setting was identified as a specific learned competency rather than a personal preference, with participants warning that failure to develop boundary management capabilities produces mental health consequences and long-term career damage. This extends the non-standard work arrangements literature (Postepska, 2022) by specifying the psychological self-management competencies required to sustain employability across hybrid and gig economy arrangements.

### Mindset competencies: cognitive demands in the 4IR

Mindset competencies (18.3% of coded references) constituted the second largest theme, encompassing critical thinking, complex problem-solving, creative and innovative thinking, decision-making and cognitive load management. The prominence of this theme relative to technology and data competencies reinforces the finding that cognitive and metacognitive capabilities are more highly valued by practitioners than purely technical digital skills, consistent with the World Economic Forum’s (2025) identification of creative thinking, resilience, flexibility and agility as the most critical future competencies, with 39% of core skills expected to change by 2030.

Critical thinking and complex problem-solving were described as having changed not in their fundamental nature but in the conditions under which they must be applied: greater speed, higher frequency, more incomplete information and more complex interdependencies. This parallels Schwab’s (2015) characterisation of 4IR velocity and depth, translating macro-level disruption characteristics into specific cognitive competency demands.

Cognitive load management and information processing emerged as a distinct sub-theme addressing the challenge of multiple simultaneous digital information streams and task-switching demands. This finding has not been adequately addressed in existing career competencies frameworks, which predate the normalisation of multi-platform, always-connected work environments. The practical manifestation described by participants, managing simultaneous demands across WhatsApp, Microsoft Teams, email and other platforms whilst maintaining focused, productive work, represents a genuinely novel cognitive competency demand that extends the intelligent careers framework beyond its original conceptualisation (Arthur et al., 1995).

### Technology and data: foundational rather than dominant

Technology and data competencies (12.1% of coded references) emerged as the smallest theme after contextual competencies, running counter to the dominant narrative positioning digital skills as the primary 4IR competency requirement (Gartoumi and Tekouabou, 2024). Participants consistently emphasised that technology and data capabilities function as foundational enablers rather than standalone career differentiators. This aligns with the intelligent careers framework’s knowing how dimension but extends it by specifying the qualitatively different nature of technological knowing in 4IR contexts: not mastery of specific tools but the ability to learn new platforms rapidly, collaborate effectively with automated systems and exercise strategic judgement about when and how to deploy technological capabilities.

Professional social media received the highest coded frequency within this theme (251 references), positioning personal digital branding as a central career competency. This reflects the shift from organisational to individual career self-management (Coetzee and Schreuder, 2018) and the growing role of digital platforms in mediating employment access (George et al., 2025). The finding extends networking literature (Beigi et al., 2018; Rehman and Qamar, 2024) by specifying that professional social media requires active curation and alignment with professional identity rather than passive presence.

AI proficiency emerged as a developing competency domain, with participants anticipating dedicated organisational roles centred on AI tool expertise. This resonates with recent evidence on AI reshaping qualification requirements and organisational structures (Bouwmans et al., 2024; Coetzee et al., 2021) and extends the knowing how dimension to encompass human-AI collaboration as a core modern competency.

### Contextual competencies: the South African specificity of the framework

Contextual competencies (11.8% of coded references) encompassed diversity and inclusion, cultural awareness, business acumen and market and business intelligence. The inclusion of this theme as a distinct competency domain represents one of the most significant differentiating features of this framework relative to Western career competency models. South Africa’s linguistic, cultural, religious and socio-economic diversity, combined with the legacies of historical inequality, creates contextual competency demands absent from frameworks developed in more culturally homogeneous settings (Laher and Cockcroft, 2018; Maree, 2019).

Cultural awareness was described as extending beyond basic sensitivity to encompass change management capabilities, sophisticated communication adaptation across digital and physical contexts, and awareness of broader societal implications of 4IR disruption. The finding that participants explicitly linked cultural competence to technological transition management adds a dimension absent from Western frameworks, which tend to treat cultural awareness and digital competency as separate domains (Sultana, 2024).

Business acumen and market intelligence reflect the intelligent careers framework’s knowing what dimension. However, contextualisation within South Africa’s high-unemployment, structurally constrained economy adds specificity absent from the original framework. Participants emphasised that business intelligence must encompass socio-economic stakeholder engagement and community-level awareness, reflecting the reality that career decisions in South Africa cannot be divorced from broader social and economic dynamics that directly constrain individual opportunity (National Planning Commission, 2012).

### Extending the intelligent careers framework

**Figure 1:**
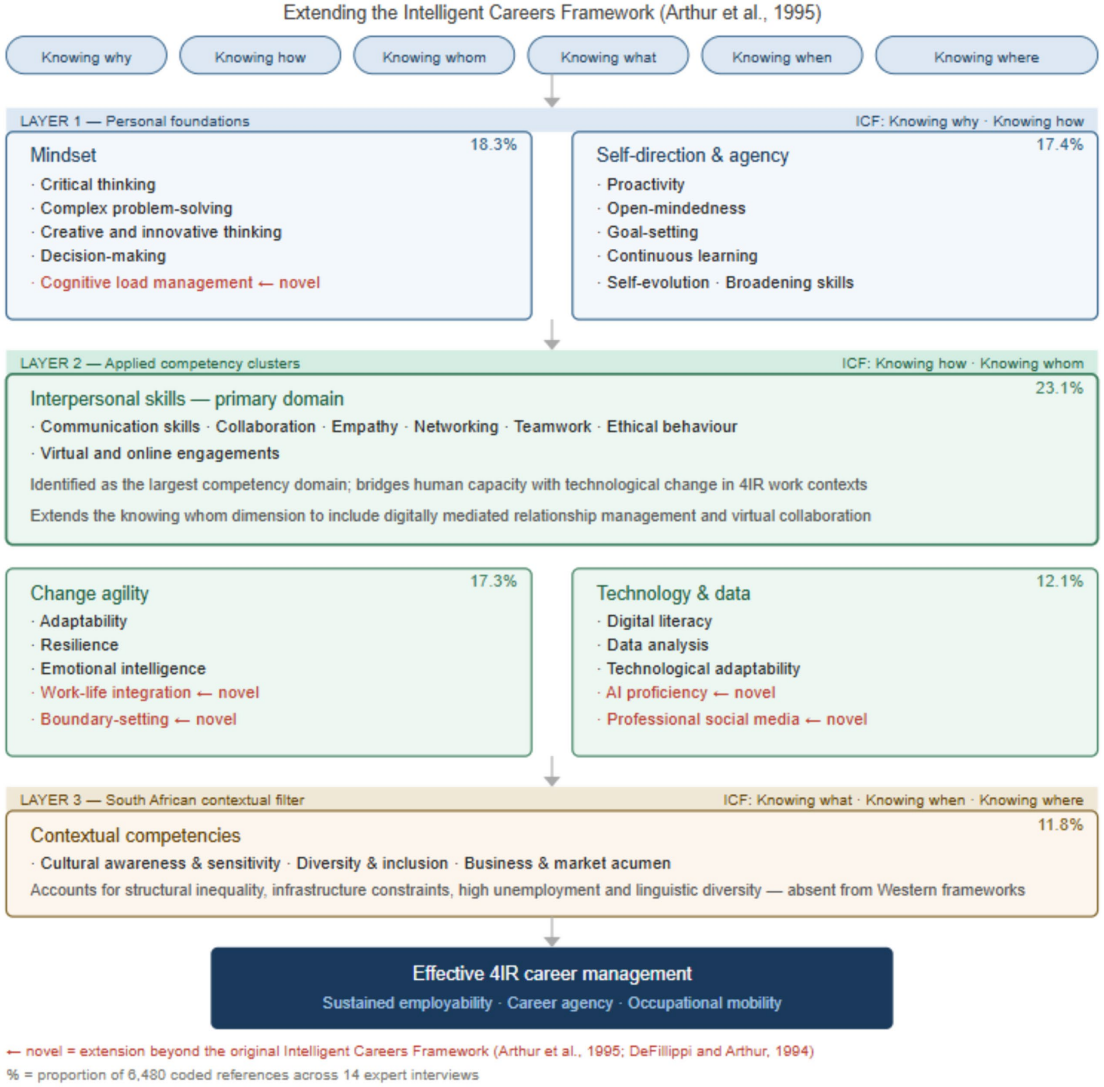
The 4IR career competency framework for South Africa.

The six-theme framework both confirms and extends the Intelligent Careers Framework (Arthur et al., 1995; DeFillippi and Arthur, 1994). Figure 1 presents the integrated framework as a three-layered model, with the Intelligent Careers Framework’s six knowing dimensions mapped across each layer to show where the study’s findings align with, and depart from, the original conceptualisation.

The first layer captures the personal foundations that individuals bring to career management. Self-direction and agency maps onto the knowing why dimension, encompassing the values, motivations and goals that drive career behaviour. Mindset competencies align with knowing how, extending that dimension beyond technical skill to include the cognitive and metacognitive capabilities and critical thinking, creative and innovative thinking, complex problem-solving and decision-making that is required for 4IR contexts. Cognitive load management and information processing is identified here as a novel sub-theme not previously conceptualised within the framework, reflecting the multi-platform, always-connected nature of contemporary work environments.

The second layer comprises the applied competency clusters through which personal foundations are enacted in workplace settings. Interpersonal skills, the largest domain at 23.1% of coded references, extends the knowing whom dimension to encompass digitally mediated relationship management, virtual collaboration and empathetic leadership in contexts of technological disruption. Change agility and technology and data competencies both extend the knowing how dimension, with two novel contributions embedded in this layer: boundary-setting and work-life integration as discrete learned competencies within change agility, and the disaggregation of technological competence into AI proficiency and professional social media within the technology and data theme.

The third layer functions as a contextual filter specific to the South African setting. Contextual competencies extend the knowing what, knowing when and knowing where dimensions by incorporating structural conditions namely, historical inequality, infrastructure constraints, high unemployment and linguistic and cultural diversity, that Western frameworks treat as background rather than as constitutive features of career development. This layer is not optional or supplementary; it shapes the conditions under which all competencies in the layers above can be developed and deployed.

Together, the three layers point toward effective 4IR career management, understood as sustained employability, career agency and occupational mobility. The framework is not offered as a fixed prescription but as an empirically grounded, contextually situated structure that is open to iterative revision as technological and labour market conditions evolve.

### The South African context: structural constraints and competency deployment

The findings must be interpreted within the structural realities of the South African context. The prominence of human-centred competencies may reflect not only their genuine importance in 4IR work but also the constraints that limit individual agency in technology-intensive domains. Where infrastructure barriers restrict digital access for substantial workforce segments (Ndung’u and Signé, 2020), interpersonal, cognitive and adaptive competencies become relatively more accessible pathways to employability. Competency frameworks for resource-constrained economies must therefore identify capabilities individuals can develop with limited institutional support alongside those requiring structural enablers.

The socio-political responses to 4IR disruption documented in this paper provide external validation that the competency gaps identified are not merely theoretical. The framework’s emphasis on change agility, self-direction and contextual competencies directly addresses the meta-competencies required to manage career transitions constructively rather than resist them reactively (Sekaja et al., 2021). Developing these competencies at scale constitutes a social stability imperative in the South African context, not merely an individual career development aspiration.

The study also confirms that Western career frameworks emphasising individual agency, values-driven choice and geographic mobility (Hall, 2002; Sultana, 2024) require fundamental reconceptualisation when applied to high-unemployment, resource-constrained economies. When career survival rather than self-actualisation is the primary motivation (Ologunoye et al., 2025), competency frameworks must address both opportunity-oriented and survival-focused capabilities simultaneously.

### Implications for practice

The findings carry practical implications for individuals, behavioural practitioners and organisations. Career proactivity (Coetzee and Schreuder, 2018; Hirschi et al., 2014), adaptability (Herrmann et al., 2015; Maree, 2012; Savickas and Porfeli, 2012), and resilience (Bimrose and Hearne, 2012; McMahon and Abkhezr, 2025; Seibert et al., 2016) are increasingly essential for sustained career success as technology continually redefines work demands. Continuous learning, skills development and the acquisition of multiple competencies are central to career progression in the 4IR (Samuel and Moagi, 2022; Zenk et al., 2024), and learning can no longer be treated as a finite process tied to a specific qualification level; it is a lifelong commitment that extends across age, seniority and professional context.

Professional networking emerged as a prominent competency dimension (Beigi et al., 2018; Rehman and Qamar, 2024). Participants emphasised that career development increasingly depends on building professional relationships across organisations and industries, facilitated by digital platforms such as LinkedIn (Blokker et al., 2019).

For behavioural practitioners, the six-theme competency framework offers an empirically grounded basis for career counselling, coaching and assessment that moves beyond generic skill lists. Career counsellors and coaches must account for how socio-economic background and access to resources shape individuals’ capacity to develop and deploy 4IR competencies, ensuring that interventions are contextually appropriate rather than assuming resource availability.

For organisations, the findings indicate that people development strategies must address both technical and interpersonal competencies simultaneously, rather than treating digital upskilling as the primary workforce priority. The prominence of interpersonal skills (23.1%) relative to technology and data competencies (12.1%) suggests that human-centred capabilities underpin effective 4IR performance and should be integrated explicitly into talent development, performance management and succession planning frameworks. Table 3 presents the framework in an applied form to support use by career counsellors, human resource practitioners and policymakers. Competency indicators are illustrative rather than exhaustive.

**Table 3:** The 4IR career competency framework for South Africa—applied reference.

Theme (% of codes)	Core competency indicators	For career counsellors and coaches	For HR practitioners and organisations
Interpersonal skills (23.1%)	Empathy; virtual communication; collaboration; networking; ethical practice	Assess relational competency alongside technical ability; include virtual communication coaching	Embed interpersonal skill development in all talent programmes; include in performance criteria
Mindset (18.3%)	Critical thinking; creative and innovative thinking; cognitive load management; decision-making	Use reflective tools to develop metacognitive awareness; address information overload explicitly	Redesign learning to include ambiguity tolerance, creative problem-solving workshops, and focus management
Self-direction and agency (17.4%)	Proactivity; goal-setting; continuous learning; self-evolution	Build individual learning plans linked to organisational needs; develop identity-level self-concept work	Invest in personal mastery programmes; reward proactive development; create psychological safety for learning
Change agility (17.3%)	Adaptability; resilience; boundary-setting; work-life integration	Include boundary-setting and digital wellness as counselling topics; build resilience through structured reflection	Introduce formal digital wellness policies; include boundary management in onboarding and EAP programmes
Technology and data (12.1%)	Digital literacy; AI proficiency; professional social media; data analysis	Include digital branding and platform literacy as career management skills	Upskill for AI collaboration, not just tool use; treat professional social media as a career asset
Contextual competencies (11.8%)	Cultural awareness; diversity and inclusion; business and market acumen	Contextualise interventions for structural constraints; address survival-oriented motivations	Build contextual intelligence into leadership development; include community stakeholder engagement

## Limitations

Four limitations should be considered when interpreting the findings. First, the sample of 14 participants, whilst appropriate for qualitative expert inquiry and consistent with information power guidance (Malterud et al., 2016), restricts the breadth of perspectives captured. Participants were predominantly highly qualified working professionals, which may not fully reflect the diverse experiences of individuals with lower educational attainment or those in precarious employment. The findings should therefore be understood as representing expert practitioner perspectives rather than the full spectrum of South African workforce experience.

Second, the study is situated specifically within the South African labour market context. Variations in economic conditions, digital infrastructure, educational systems and cultural dynamics across other regions or countries could produce different competency priorities. The findings are not directly transferable to other national contexts without adaptation.

Third, as with all interview-based research, social desirability bias may have influenced participants’ responses, with individuals potentially presenting competencies in a more favourable or professionally acceptable light than their lived practice reflects (Podsakoff et al., 2003). Whilst member checking and reflexive journalling were employed to mitigate this risk, it cannot be eliminated entirely.

Fourth, the sample was predominantly female (71.4%), which was a function of purposive sampling based on expertise and availability rather than intentional design. Gender may shape career experiences and perceptions of competency demands, particularly in the South African context where gendered labour market inequalities remain pronounced. The perspectives of male practitioners and those in non-managerial roles are consequently underrepresented, and future research should deliberately recruit across gender to examine whether competency priorities differ.

### Recommendations for future research

Several directions for future research emerge from this study. Future studies should expand both sample size and geographic scope to include participants from all South African provinces and from a broader range of occupational levels, including those with limited formal qualifications or in precarious employment, which would strengthen the representativeness of the findings and improve their applicability to the wider South African workforce.

Quantitative or mixed-methods research should be pursued to validate the six-theme competency framework identified in this study. Survey-based approaches could test the framework across large, representative samples, enabling statistical examination of how competency priorities vary by sector, educational level, age group and region (Creswell and Plano Clark, 2018), moving the framework from an expert-derived construct to an empirically validated instrument.

Longitudinal research is needed to track how 4IR competency requirements evolve as technologies such as generative artificial intelligence, automation and platform-mediated work continue to mature. Cross-sectional findings capture a moment in time, but the pace of technological change means that competency frameworks require systematic review and updating (World Economic Forum, 2025).

Future research should also examine the intersection of structural barriers and individual competency development more directly. Studies exploring how infrastructure constraints, unemployment and rigid certification systems specifically limit competency acquisition and deployment would provide actionable evidence for targeted policy and organisational intervention (Gartoumi and Tekouabou, 2024; Ndung’u and Signé, 2020). Finally, comparative studies across African economies at different stages of 4IR adoption would contribute to more contextually nuanced theoretical frameworks that move beyond assumptions of Western applicability (Ologunoye et al., 2025; Sultana, 2024).

## Conclusion

This study set out to establish career competencies for effective career management in South Africa’s 4IR workforce and to elicit expert insights on emerging competency demands. Thematic analysis of 14 expert interviews yielded 6,480 coded references across six competency themes and 28 sub-themes, producing a contextually grounded framework that extends and operationalises the intelligent careers framework (Arthur et al., 1995; DeFillippi and Arthur, 1994) for 4IR conditions in a developing economy.

The most consequential finding is that interpersonal skills constituted the largest competency domain, substantially exceeding technology and data competencies. This challenges the technology-dominant assumptions that have shaped much of the 4IR workforce literature and reinforces the growing body of evidence that human-centred capabilities, particularly empathy, collaboration and virtual engagement, are the primary differentiators of 4IR performance (World Economic Forum, 2021). Mindset competencies, including critical thinking, creativity and cognitive load management, ranked second, further supporting the view that metacognitive and interpersonal capacities underpin technological competence rather than the reverse.

Three substantive theoretical contributions emerge from this study. First, cognitive load management and information processing are identified as a discrete career competency not previously conceptualised in career frameworks, responding to the multi-platform, always-connected nature of contemporary work. Second, boundary-setting and work-life integration are established as specific learned competencies requiring deliberate development, extending the self-management literature beyond generic adaptability to address the psychological demands of digitally mediated work. Third, the inclusion of contextual competencies as a distinct domain reflects the structural realities of South African working life, including racial and economic inequality, uneven digital infrastructure and high unemployment, which Western frameworks treat as background conditions rather than constitutive features of career development.

The framework also has relevance beyond the 4IR context. The Fifth Industrial Revolution (5IR), which explicitly recentres human values and well-being within technological systems (Maddikunta et al., 2022), finds direct antecedents in the competencies identified here. The prominence of empathy, virtual collaboration and boundary-setting in a study focused on 4IR demands indicates that practitioners are already navigating a transition towards the human-centred paradigm that the 5IR formalises. This gives the framework enduring utility as both a 4IR readiness tool and a foundation for 5IR workforce development.

The framework is not offered as a definitive inventory but as an empirically derived, theoretically coherent foundation for further development. Its utility for practice is immediate: career counsellors and coaches gain a contextually appropriate basis for competency-focused interventions; human resource practitioners gain a framework for talent development that treats interpersonal and cognitive competencies as primary rather than supplementary; and policymakers gain evidence-based guidance for workforce development strategies that account for structural constraints rather than assuming resource availability.

## Data Availability

The raw data supporting the conclusions of this article will be made available by the authors, without undue reservation.
